# Tips and Tricks for Navigating the Microvascular Coupler Anastomosis Learning Curve

**DOI:** 10.1055/a-1988-3066

**Published:** 2023-02-07

**Authors:** Juan Enrique Berner, Georgios Pafitanis, Dariush Nikkhah, Timothy P. Crowley, Maniram Ragbir

**Affiliations:** 1 Department of Plastic Surgery, Emergency Care and Trauma Division (ECAT), The Royal London Hospital, Barts Health NHS Trust, London, United Kingdom; 2 Plastic Surgery Department, Royal Free Hospital, Royal Free London NHS Foundation Trust, London, United Kingdom; 3 Plastic Surgery Department, Royal Victoria Infirmary and Freeman Hospital, The Newcastle upon Tyne Hospitals NHS Foundation Trust, Newcastle, United Kingdom

Microvascular anastomotic couplers systems (MACS) have gained wide acceptance among reconstructive microsurgeons, demonstrating reliability and efficiency in venous anastomosis. 1 The GEM/MACS (Synovis, USA) has two components: A reusable titanium coupler applicator device and a single-use coupling device that holds the premounted microvascular rings, in a book-like rectangular plastic leaf held with a spring.

When performing end-to-end microvascular anastomosis with the MACS device, the first step is to measure the caliber of the donor and recipient vessels using the measuring gage provided. The MACS has two opposing plastic rings with interdigitating metal spikes, into which the veins are passed, everted, and pulled over the spikes to be held in position. Once this step is complete, the handle at the proximal end of the applicator is turned, bringing the plastic rings together, closing the device, and the interdigitating metal spikes. The ring is then pushed out of the device, delivering the anastomosed vessels.

Even though using the MACS for end-to-end microvascular anastomosis is easy and reproducible, it involves a learning curve. 2 In our experience with the MACS device over a decade of frequent use, we have identified several challenges and troubleshooting techniques associated with the hardware of this technology. Here, we describe five technical tips that can enhance efficiency when performing end-to-end anastomosis with the MACS for microsurgeons who may be getting familiarized with this technology.

**Fig. 1:**
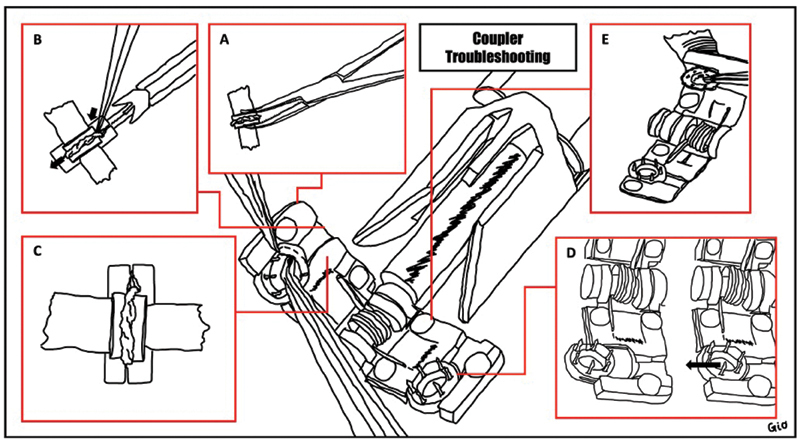
Diagram illustrating the five tips presented in this article. ( **A** ) A fine mosquito forceps can be used to secure the rings by approximating them further. ( **B** ) Rings can be pushed out of the device if the applicator gets jammed while delivering the anastomosis. ( **C** ) Adequate preparation helps avoid having soft tissues trapped in the device. ( **D** ) If the rings fall off the device, they can be carefully reinserted. ( **E** ) For 1 and 1.5 mm couplers a pair of fine-curved vessel dilators is useful to secure the vein on to the metal spikes.

Tip 1: *Securing ring closure* . As the coupler device is being operated and the plastic rings come together, the force frequently does not close the rings fully. In that circumstance, most microsurgeons would use a pair of mosquito forceps to obtain a tight closure of the rings by gently approximating and securing them ( Fig. 1A ).

Tip 2: *Safely delivering the anastomosis* . Sometimes once the rings are closed the coupler applicator may jam and fail to push the rings out. This is a difficult situation when dealing with small or friable vessels, or when working in restricted spaces. We would advise in this scenario that it is best not to persevere, as excessive force applied to the applicator may result in inadvertent movement that may compromise the anastomosis. While controlling the applicator, the closed rings can be gently pulled out with a pair of mosquito forceps. If this is not possible, a pair of microsurgical forceps can be inserted between the rings and the rest of the coupling system, allowing the rings to be pushed out rather than pulled ( Fig. 1B ). Following an episode like this, the coupler applicator should be sent for inspection.

Tip 3: *Dealing with surrounding adventitia and soft tissues* . It is not infrequent that vessel adventitia or surrounding soft tissues get trapped in the coupling device. This can be avoided by trimming the adventitia next to the anastomosis and removing any redundant tissues around the pedicle in the 10-mm next to the planned anastomosis, as this provides enough space for the coupler applicator to operate ( Fig. 1C ). Care should be taken when dealing with small veins as excessive tissue manipulation can result in spasm and vessel injury. If despite adequate preparation tissues still get trapped in the coupler device, the anastomosis can still go ahead. Once the rings have been delivered it is best not to pull the applicator out, but to turn its handle in the opposite direction. Once the coupler device opens back again, all soft tissues are automatically released from the system.

**Fig. 2:**
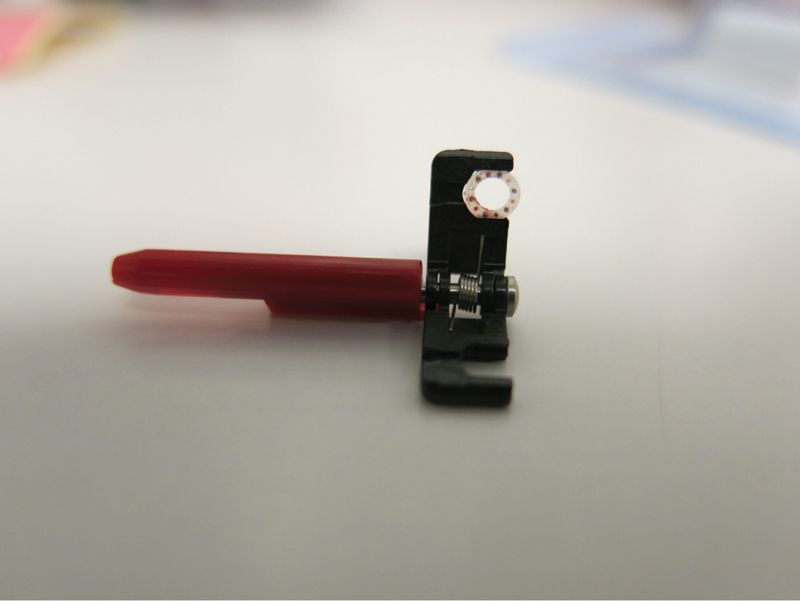
Clinical picture demonstrating how one of the coupler rings can fall off the coupler device while attempting an anastomosis.

Tip 4: *Remounting rings on coupler device* . Rarely, one of the coupler rings may fall off the coupling system ( Fig. 2 ). This can be very distressing when performing a difficult anastomosis, especially if one ring has been mounted already. In this situation, the plastic ring can be gently remounted in the coupler system. These rings have rail marks that allow remounting in a precise manner, maintaining its original position and allow correct interdigitation with the metal spikes of the opposing ring ( Fig. 1D ). If, however, any of the metal spikes becomes damaged, such as bent or loosened, we would then recommend that the device is discarded and a new coupler used. Any closure of the device without correct alignment of the metal spikes risks inadvertent closure of or damage to the anastomosis.

Tip 5: *Sizing couplers and dealing with a small vein* . The coupler anastomosis is easier to perform if the vessels are the same size or slightly larger than the chosen coupler, as overstretching veins can lead to their wall tearing. For the same reason, when dealing with mismatch we tend to choose the coupler size to fit the smaller vein. If a coupler smaller than 1.5 mm is required, fixing the vein to the metal spikes can prove challenging as the space between the spikes is small. If the provided pair of “hockey stick” forceps is too big for this task, a pair of curved vessel dilators may be more appropriate, allowing to push the vein walls flush in restricted spaces ( Fig. 1E ).

MACS have become a standard of care for venous anastomosis. In experienced hands, this device improves efficiency and shortens operative times. We believe that the abovementioned tips can enhance troubleshooting and increase young microsurgeons' familiarity with this technology.
